# Physical activity interventions in women treated for endometrial cancer: a scoping review

**DOI:** 10.1007/s11764-025-01938-7

**Published:** 2025-11-28

**Authors:** Leanne Shearsmith, Hannah Truscott, Abigail Fisher, Gemma Traviss-Turner, Emma J. Crosbie, Andrew Hill, Rebecca Beeken

**Affiliations:** 1 Leeds Institute of Health Sciences, University of Leeds, Leeds, UK; 2 Department of Behavioural Science and Health, University College London, London, UK; 3 Department of Obstetrics and Gynaecology, St Mary’s Hospital, Manchester University NHS Foundation Trust, Manchester Academic Health Science Centre, Manchester, UK; 4 Division of Cancer Sciences, University of Manchester, Manchester, UK

**Keywords:** Endometrial cancer, Physical activity interventions, Exercise oncology, Cancer survivorship, Quality of life, Scoping review

## Abstract

**Purpose:**

Endometrial cancer is the most common gynaecological cancer, with rising incidence contributing to a growing population at risk of long-term health issues post-treatment. Cardiovascular disease is the leading cause of mortality, yet physical activity engagement is low due to physiological and psychosocial barriers. Although guidelines recommend physical activity after cancer, no reviews have synthesised interventions supporting uptake in women treated for endometrial cancer. This review addresses the gap.

**Methods:**

A mixed-methods scoping review was conducted. Medline, EMBASE, Cochrane, Web of Science, PEDro, EThOS, and PsycInfo were systematically searched for studies published from January 2011 to October 2024. Eligible studies recruited adults post-treatment for endometrial cancer and reported interventions supporting physical activity.

**Results:**

Twenty-two papers describing 12 interventions met inclusion criteria. Six (50%) focused on physical activity only, three (25%) combined diet and physical activity, and three (25%) were multicomponent interventions incorporating diet, physical activity, and additional elements such as mindfulness. Most studies were USA-based (77%). One powered RCT and nine pilot or feasibility trials were included. The RCT reported a significant increase in total physical activity minutes post-intervention. Most studies reported improvements in quality of life, physical function, and self-efficacy. Adherence ranged from 36% to 86%, facilitated by social interaction and hindered by pain, fatigue, and caregiving responsibilities.

**Conclusion:**

Early-stage evidence suggests physical activity interventions are feasible in this population.

**Implications for Cancer Survivors:**

Even modest increases in physical activity may improve quality of life. Future research should integrate co-design, cultural adaptation, and tailoring interventions to baseline mental health, BMI, and activity levels.

**Supplementary Information:**

The online version contains supplementary material available at https://doi.org/10.1007/s11764-025-01938-7.

## Introduction

Endometrial cancer is the most common gynaecological cancer in developed countries, with incidence rates rising over the past three decades [1] Global prevalence currently exceeds 1.4 million cases [2] Incidence is projected to rise by 60.9% by 2050, reaching over 676,000 new diagnoses per year [3] Advances in treatment have improved survival rates, resulting in a growing population of women living with and beyond endometrial cancer [4] However, these individuals face considerable long-term health risks, with mortality rates up to 16 times higher than those observed in age-matched women in the general population [5, 6] Cardiovascular disease is the leading cause of mortality in women treated for endometrial cancer [7, 8], so improving and maintaining cardiovascular health is a critical component of endometrial cancer survivorship care. Physical activity is one way to achieve this [7].

The benefits of physical activity for cardiovascular health are well established [9], and observational data suggest that among women treated for endometrial cancer, higher levels of post-treatment physical activity are associated with a 20–30% reduction in cardiovascular disease [7] Physical activity is associated with reduced risk of cancer recurrence [10] improved disease-free survival and overall survival [11], better quality of life [12], and reductions in cancer-related fatigue [13].

The safety and feasibility of physical activity after a cancer diagnosis is supported by a large body of trial evidence, which demonstrates improvements across many physical and psychosocial outcomes [14–16]. While evidence shows that physical activity is important across all stages of the cancer continuum, physical activity in the survivorship phase can: 1) manage treatment toxicities, 2) improve physical function, 3) improve psychosocial outcomes, and 4) support quality of life [17]. However, very few trials to date have focused on women treated for endometrial cancer. A 2023 scoping review of exercise interventions across all gynaecological cancers identified only three studies in endometrial cancer: one pilot RCT, one wait-list controlled feasibility trial, and one single-arm pre-post study [18]. All three studies reported that home-based physical activity interventions were feasible and demonstrated preliminary improvements in physical activity behaviour, quality of life, and physical function. The review concluded that women treated for endometrial cancer are at greater risk of challenges to physical quality of life and emphasised the need to support exercise in this population.

Women who have completed primary treatment for endometrial cancer face a distinct set of physiological and psychosocial barriers to physical activity. In addition to common cancer-related symptoms such as pain, neuropathy, and fatigue [19], women report adverse effects specific to gynaecological cancer treatment. These may include iatrogenic menopause, lower limb lymphedema, and urinary dysfunction [20–22], all of which can reduce motivation or perceived capability to engage in physical activity. Psychosocial barriers include uncertainty around safe frequency, intensity, and types of physical activity; obesity-related stigma causing fear of judgement when exercising in public or gym spaces; and insufficient information or support from healthcare professionals [23]. Systematic review evidence shows a consistent decline in physical activity across the gynaecological cancer survivorship journey, with 91% falling below cancer-specific physical activity guidelines of ≥ 150 min moderate-intensity aerobic training and ≥ 20 min resistance training per week [24]. Physical activity levels in women with endometrial cancer are lower than in other cancer populations, with an observed median daily step count of just 1,309, compared to women with breast cancer (7,409 steps per day) [25]. Population-level survey data show that this population is more likely to be inactive, to live with obesity, and to have lower household incomes (below $25,000) [26]. Qualitative data help explain these disparities. Women treated for endometrial cancer describe financial barriers for accessing support, resources and opportunities, feelings of shame and judgement in public spaces such as gyms, and limited communication from healthcare providers regarding physical activity [27]. These intersecting and compounding barriers underscore the inadequacy of one-size-fits-all approaches and highlight the need for tailored, supportive, and accessible interventions.

Racial and systemic inequities further shape outcomes in endometrial cancer; mortality rates are disproportionately higher in women from Black ethnic backgrounds (80–90%) [28, 29]. Minoritised ethnic groups face systemic barriers to accessing healthcare, which contribute to disparities in care [30]. Qualitative research has also identified inequities in patient information and support resources, with Black women reporting insufficient cultural sensitivity, representation, and language accessibility [31]. Culturally tailored interventions may be particularly valuable in this context, as they adapt intervention design, delivery, and content to align with the cultural needs and preferences of specific populations [32]. The inclusivity of health interventions can also be improved by incorporating the lived experiences and priorities of the target population through methodologies such as co-design, in which patients, caregivers, and community partners actively collaborate with researchers to develop interventions [33]. Grounding interventions in behavioural theory has also been suggested as a way to improve their effectiveness [34].

Despite growing recognition of the importance of physical activity for women treated for endometrial cancer, to our knowledge few reviews have focused specifically on this population. One systematic review evaluated weight-loss interventions (including physical activity components) but was limited to RCTs and focused on clinical outcomes such as survival and adverse events in women living with overweight and obesity [35]. One previous systematic scoping review examined the efficacy of exercise interventions across all gynaecological cancers (ovarian, peritoneal, endometrial and cervical cancers) [36]. While valuable, the review included exercise-only intervention studies, thus excluding multicomponent lifestyle interventions. Examining lifestyle interventions that include physical activity components alongside diet or psychosocial support may illuminate approaches that could be beneficial. The current review is novel in its approach of including mixed-method study types (quantitative, qualitative, and observational), capturing all available evidence on physical activity in women who have completed primary treatment for endometrial cancer. This focus reflects the growing need to support physical activity in the post-treatment phase of survivorship, when the focus shifts from treating the diagnosis to managing long-term health and late effects of treatment. The end of treatment is a time when patients report being receptive to making lifestyle changes [37].

The value of scoping reviews to evidence-based practice is in their ability to map diverse bodies of literature and identify gaps in research, determine the extent of the research available, and how it has been conducted [38]. In this context, a systematic scoping review approach is ideal for examining the variety of physical activity interventions and the methodologies used, assessing whether any studies have targeted underserved populations, and providing a comprehensive overview to guide future research and intervention development. This systematic scoping review aims to determine the extent, range, and nature of the existing literature on physical activity interventions for women after treatment for endometrial cancer.

## Methods

The scoping review protocol was registered prospectively with the Open Science Framework on September 20, 2023 [39]. The scoping review was conducted in accordance with the framework developed by Arksey and O’Malley [40] in the following stages: 1) Identifying the research question and objectives, 2) Identifying relevant studies, 3) Study selection, 4) Data extraction and charting the data, 5) Collating, summarising and reporting the data. Further in line with this methodology, the quality of evidence for included studies was not assessed.

### Data sources and search strategy

A search of the literature was conducted in 7 online databases: Medline, EMBASE, Cochrane, Web of Science Core Collection, PEDro, EThOS, and PsycInfo (Appendix 1). The search included publications from 01 January 2011 to October 2023 and was updated in October 2024. The lower date boundary follows the publication of key exercise guidelines for individuals diagnosed with cancer [41], and although there are no specific guidelines for endometrial cancer survivors, the emergence of guidelines underscores a significant shift in attention towards exercise oncology research. In addition, this period captures a growing body of literature influenced by improved treatment outcomes and survivorship rates in endometrial cancer, along with increasing awareness of comorbidity risk and the role of physical activity in survivorship care. The search strategy was developed with advice from an Information Specialist using a combination of subject headings (e.g. MeSH), and additional key words derived from scoping searches and input from a Professor of Gynaecological Oncology (EJC) (see Appendix 1 for search strategy). These databases were searched for relevant published articles and grey literature (such as dissertations, conference abstracts and research reports). Authors were contacted in cases where relevant trial registrations or conference abstracts were found but full texts were not available.

### Study selection

Quantitative, qualitative and mixed-methods studies were included in the review. The search results were uploaded into Rayyan [42], a literature management and research collaboration platform, for duplicate removal and screening. After removing duplicates, two authors (LS and HT) independently screened titles, abstracts, and full texts in three stages. Entries were coded as ‘include’, ‘exclude’ or ‘maybe’. Full-text articles were analysed to confirm their eligibility based on the predefined inclusion criteria. For citations that did not meet the inclusion criteria, the reason for exclusion was documented in accordance with the PRISMA-ScR statement.

The inclusion criteria were developed in line with the JBI Person, Concept, Context guidelines [43], and applied as follows: 1) studies including adult participants aged 18 years and older with a diagnosis of endometrial cancer who had completed primary treatment with curative intent, 2) studies describing an intervention aimed at maintaining or increasing physical activity in women with endometrial cancer (or an intervention with at least one physical activity component), and 3) studies reporting the experiences of women with endometrial cancer participating in a physical activity intervention. All intervention contexts and delivery methods were considered, including home-based programmes, in-person sessions, and community settings. Studies employing mixed samples of participants undergoing and post-treatment, where these groups were not analysed separately, were excluded. No restrictions were placed on study design; all methodological approaches were eligible for inclusion. Studies were not selected based on outcomes; both objective measures and subjective reports of physical activity and health were included. The search was not limited to English language publications.

Exclusion criteria were as follows: 1) studies involving non-human subjects and 2) opinion articles. During the initial phase of screening by title and abstract, two additional exclusion criteria were introduced: 3) studies where the participant sample did not include at least 50% endometrial cancer patients, and 4) interventions focused on pelvic floor muscle training. The first criterion was introduced to ensure that the findings would be relevant and specific to endometrial cancer. The second criterion was added because pelvic floor muscle training, which involves the repetition of voluntary contractions of the pelvic muscles to strengthen the bladder, rectum, and uterus, is technically categorised under 'physical activity’, but is a specific physiotherapy technique with a distinct clinical purpose of reducing genitourinary symptoms, which did not align with our research aims.

### Data extraction

Two authors (LS and HT) independently extracted data from each article using the JBI data extraction instrument for scoping reviews: Author(s); Year of publication; Origin/country of origin (where the source was published or conducted); Aims/purpose; Population and sample size within the source of evidence; Methodology; Intervention type, comparator and details of these; Duration of the intervention; Outcomes and details of these (e.g. how measured); Key findings that relate to the scoping review question [44]. Modifications were made as outlined in the protocol to include additional data relevant to the review question: sample characteristics (ethnicity and percentage of the sample with an endometrial cancer diagnosis), outcome measures used, and conclusions from the authors. Authors (LS and HT) were blinded to each other’s data extraction; then once complete, data extraction tables were compared for accuracy and for any cells where data differed, the data was checked against the published paper and cell contents merged.

### Quality appraisal

In line with guidance for scoping reviews [43, 45], a formal assessment of methodological quality or risk of bias was not conducted, as the aim was to provide an overview of the existing evidence regardless of quality.

## Results

### Study selection

**Fig. 1:**
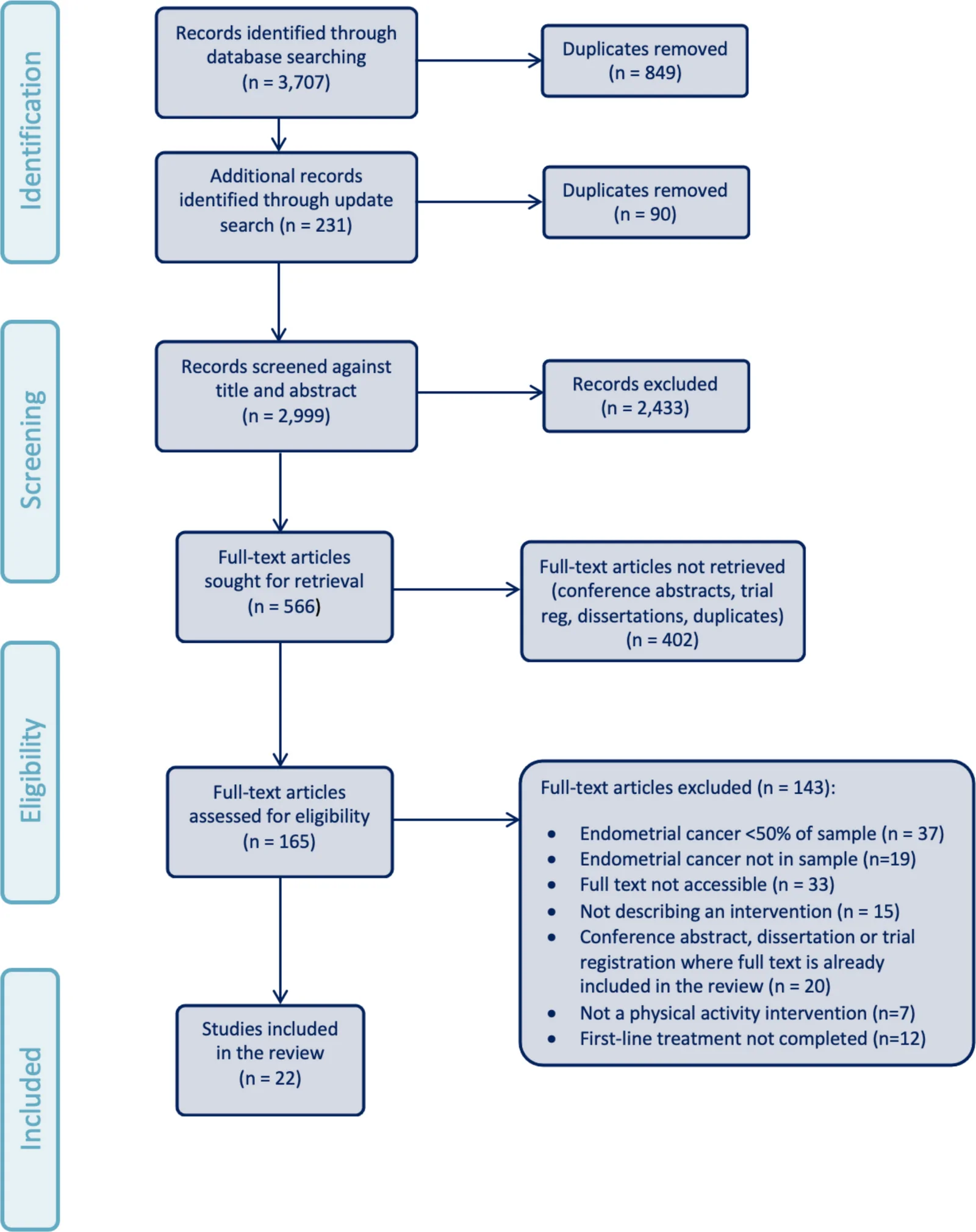
A PRISMA flow diagram

Figure 1 presents the PRISMA flow diagram. A total of 3,707 records were identified from 7 electronic databases. After removing 849 duplicate records, 2,858 records remained. Following title and abstract screening, 2,633 records were excluded and 553 were sought for retrieval of full texts. Of these, 402 were not retrieved because they were conference abstracts or trial registrations without associated full publications, or where the full study was already included elsewhere in the review. Where full texts were not available, 7 authors were contacted, resulting in 1 additional record retrieved. An updated search in October 2024 identified 231 further records. After removing 90 duplicates, 141 records were screened. Following title and abstract screening, 128 were excluded, 13 full texts were assessed, and 1 new eligible record was identified.

In total, 165 full texts were assessed for eligibility, and 143 were excluded (Fig. 1 presents reasons for exclusion). Twenty-two studies met eligibility criteria and were included in this scoping review.

### Study characteristics

An overview of study characteristics is presented in Table 1. Twenty-two papers describing 12 interventions were included, with some interventions reported across multiple publications, including protocols, primary results, and secondary analyses. Studies were published between 2011 and 2024. Early publications (2011–2015) were relatively few, with an increase in output from 2016 onward, peaking in 2021 with 5 studies and continuing into 2022–2024. The geographical distribution of papers was predominantly USA-based (n = 17), with smaller representation from the United Kingdom (n = 4) and Hong Kong (n = 1).

**Table 1:** Overview of included interventions supporting physical activity engagement in women treated for endometrial cancer

Intervention/author name	Intervention content	Format	Intervention materials/equipment	Duration	Intervention tailoring?
Physical activity only					
Moving Away from Cancer	• Aerobic activity (30 minutes daily walking) and strength-training exercises (exercise bands) • 1 month of weekly telephone coaching calls, 5 months biweekly phone calls	Telephone based	• Workbook with instruction materials • Exercise log • A pedometer • Exercise instruction/reminder magnet	6 months	No
STEPS to HEALTH	• Individual exercise prescription • Gradually increasing moderate-intensity walking (goal of achieving 30 min of accumulated activity on most days of the week) • Telephone counselling for activity adherence	Telephone based	• Written materials	6 months	Customised exercise prescription based on participants’ baseline fitness
Rossi	• Twice-weekly intervention sessions: group counselling (30 mins) focused on improving exercise self-efficacy. Supervised exercise session (60 mins) including bodyweight resistance exercises and cardiovascular fitness training	Combination of in person group counselling & exercise, and individual, home-based walking	N/A	12 weeks	Yes, tailored for sociocultural diversity: • Class instructor was a Black Dominican woman & native English and Spanish speake • Musical choices & dance steps modified with participant input • Individually tailored walking maps, greater emphasis on high-density areas
EPEC-FAST	• Weekly 60-minute training sessions with a personal trainer. • Each session consisted of: 10 min warm-up 40 min workout of a combination of aerobic exercise (cardiovascular), pillar strength exercise (including hip and core stability) and resistance training 10 min cool down	Individual (one-to-one) supervised exercise programme In person at a local gym facility	• Participants received general physical activity recommendations for moderate intensity exercise for 150 min per week	10 weeks	• Tailored to the individual patient through a health assessment which took into consideration their current health status, physical activity level, comorbidities and medical history.
Fitbit programme	• Initial exercise consultation • Randomised to receive reminders and goal-setting counselling via telephone communication or to receive electronic reminders via email or text. • Communication was every 2 weeks for 2 months then once during months 4 & 5.	Home-based (technology intervention with telephone or email/text reminders to walk)	• A Fitbit Alta (instructed to wear the Fitbit continuously except for sleep and waterrelated activities	5 months	No, but gradual approach to increasing PA was taken (intervention encouraged a goal of step count increase in 10% increments)
Gorzelitz	• Home-based strength exercise performed twice-weekly for 10 supervised week • 5 weeks of follow-up	Telehealthdelivered home-based exercise intervention	• Educational materials • Exercise equipment • Support/feedback via video coaching sessions	10 weeks	No
Diet and physical activity					
DEUS	A total of 8, weekly 90-minute sessions facilitated by a research dietitian following a standardised scripted manual. Sessions focused on: • Regular eating pattern, food balance & portion sizes • Reducing sedentary activity, increasing lifestyle and organised activities • Behavioural self-monitoring & goal setting, problem solving	Group based (face-to-face in hospital setting)	• “Shape-Up following cancer treatment” manual & booklet with healthy lifestyle advice after study completion	8 weeks	No
SUCCEED	A total of 16 (10 weekly and 6 bi-weekly) 60- minute sessions on: • Nutrition (improving diet quality, portion sizes, meal planning, food labels & social eating) • Physical activity (walking goals of 10,000 steps/day or an increase of 2000 steps/day from baseline, encouragement to adopt activity they enjoyed to meet overall group PA goal of 150 min/week for months 1–2, 225 min/week for months 3–4 and 300 min/week (5 times/week for 60 min) for months 5–6 • Physician counselling visits at 3, 6, and 12 months conducted on 1:1 basis for overall health concerns, review of medications and co-morbidities.	Group based and individual, face-to-face	• Weekly food/activity records • Scales to weigh participants before each session • Newsletters to encourage adherence after intervention ended (content included seasonal recipes and ways to increase PA) • Pedometer • Hand & ankle weights 3lbs with instructions for performing resistance exercises. • Heart rate monitors for target heart rate goals.	6 months	No
Schwartz	• Physical activity: target recommendations of 10,000 steps per day and 25–30 minutes moderate intensity walking 5 times/week • Nutrition: target recommendations of daily caloric intake • Participants contacted every few weeks to provide support and identify safety concerns	Home-based	• FitBit activity monitor • Exercise diary	12-weeks	Caloric intake was personalised based on adjusted body weight to achieve weight loss
Lifestyle interventions					
Mindfulness in Motion + Dietary counselling (MIM+D)	A total of 8 weekly mindfulness-based sessions: • Mindful yoga - movements and breathing exercises for stress management • Mindful eating - education and skills around nutrition (energy balance, portion sizes, macronutrients, meal planning) and behaviour change (SMART goal setting, self-monitoring, and motivation) 6 weeks of home-based practice	Combination of face-to-face and home based	• CD/DVD and guidelines for homebased mindfulness and yoga	14 weeks	No
WWACPHK (Women’s Wellness After Cancer Programme)	• 3 virtual consultations with trained research nurses to provide advice on the development & tailoring of action plans, goal setting, and motivational interviewing to encourage adherence to participants’ action plans • Dedicated study website for the selfmonitoring of dietary habits and physical activity • eBook with information about healthy diet, exercise, menopause, sleep, sexuality, body image, pelvic floor exercises, stress management, chronic disease prevention & health screening	Online	• electronic book (eBook) with health information on chronic disease prevention • an interactive website • online discussion forum facilitated by a trained research nurse	12 weeks	This is an adapted version of “Women’s Wellness after Cancer Program (WWACP)” developed for Australian women with breast, gynaecological & blood cancers. Adaptation for Chinese women involved: • Healthy diet recommendations modified to fit within a Cantonese diet • eBook translated into Traditional Chinese • Removal of information about skin cancer • Healthy body mass index (BMI) range for Asians • Ethnically matched role models - added video clips involving a Chinese woman demonstrating muscle strengthening and balance exercises
SUCCEED (adapted version for African American women with EC)	• A total of 12 weekly sessions of supervised physical activity (warm-up, 25 min of dance fitness, 20 min of resistance training using body weight and elastic exercise bands, a cool-down, and stretching) • Home-based target of 90-minutes/week of moderate intensity walking between sessions • Written materials providing nutritional counselling/education (healthy recipes, food groups, reducing intake of simple sugars and concentrated fats, food labels)	Combination of face-to-face group and individual home based	• FitBit activity monitor • Educational written materials (meal planning, uterine cancer educational information, portion sizes, social eating) • Diet & physical activity record	12 weeks	Yes, intervention tailored to African American women through: • Culturally tailored recipes • Exercise routine and musical playlist based on participant feedback • Fitbit app/website interface allows socialising with friends, and group challenges • Oversight from College of Nursing members on cultural appropriateness of data collection & intervention materials

**Fig. 2:**
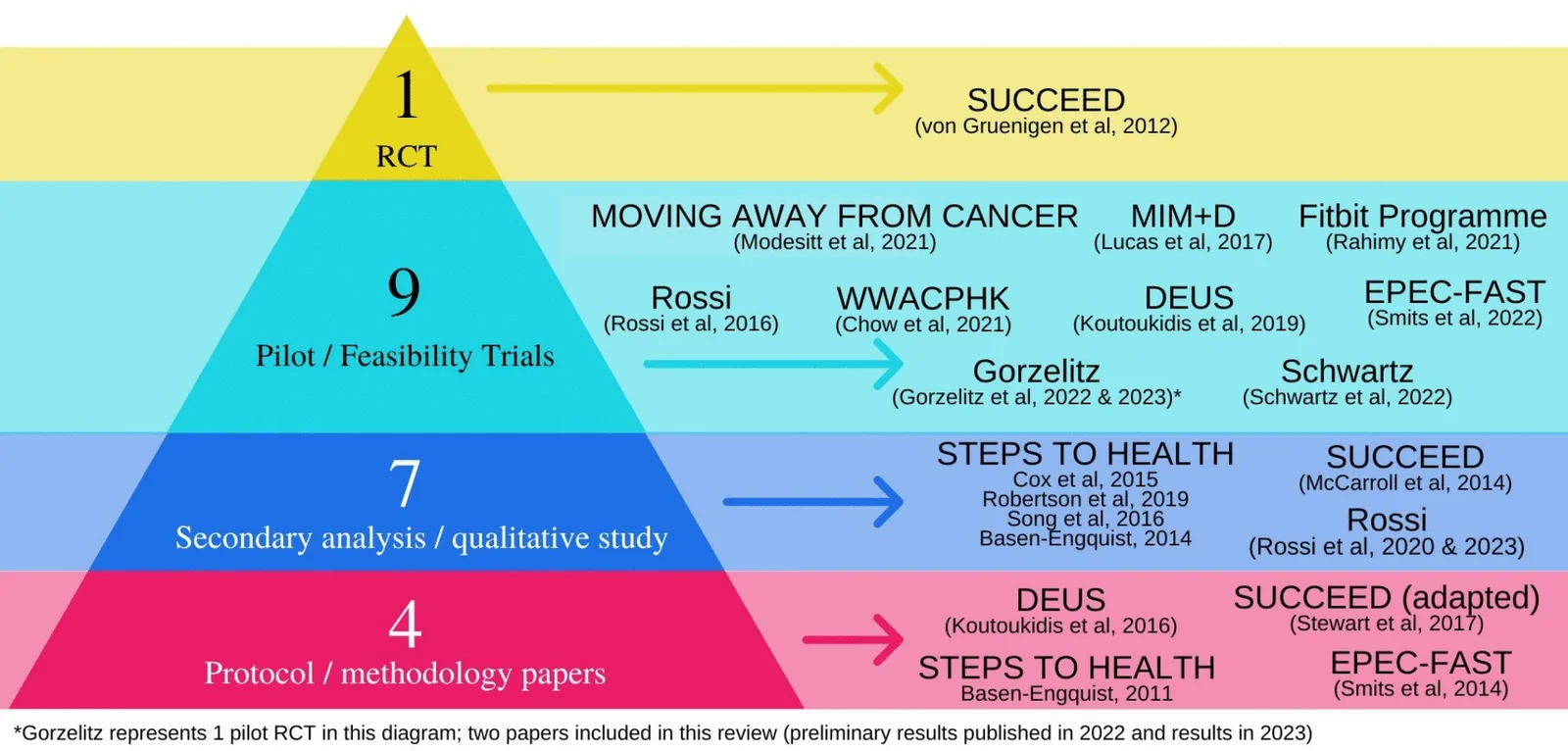
A diagram showing the trial types of included studies

Figure 2 presents the range of evidence types. One fully powered randomised controlled trial (RCT) was included. Early-phase evaluation was evident in 4 pilot RCTs, 1 single-group pre-post pilot, and 4 feasibility studies, including one non-powered phase II RCT. One embedded qualitative study explored participant perspectives and intervention engagement [46]. Three protocols corresponding to pilot and feasibility trial results were included, although 1 culturally adapted intervention protocol (published in 2017) had not reported results at the time of review [47]. One methodology paper detailed the intervention development process [48]. Six secondary analyses extended understanding of intervention effects by investigating behavioural mechanisms such as self-efficacy and outcome expectations, analysing changes in health-related quality of life, and assessing the impact of obesity status on intervention responses. Most studies employed quantitative methods; 3 incorporated qualitative evaluation within mixed methods designs.

### Sample characteristics

Across the 22 studies, 483 participants were included, with sample sizes ranging from 9 to 99 (average n = 44, median n = 28). Nineteen articles included women treated for endometrial cancer only. Three articles included mixed gynaecological cancer samples (endometrial, ovarian, cervical and “other”) with endometrial cancer representing 54–58.6% of samples. These studies reported outcome data for the full sample without stratification by cancer type.

**Fig. 3:**
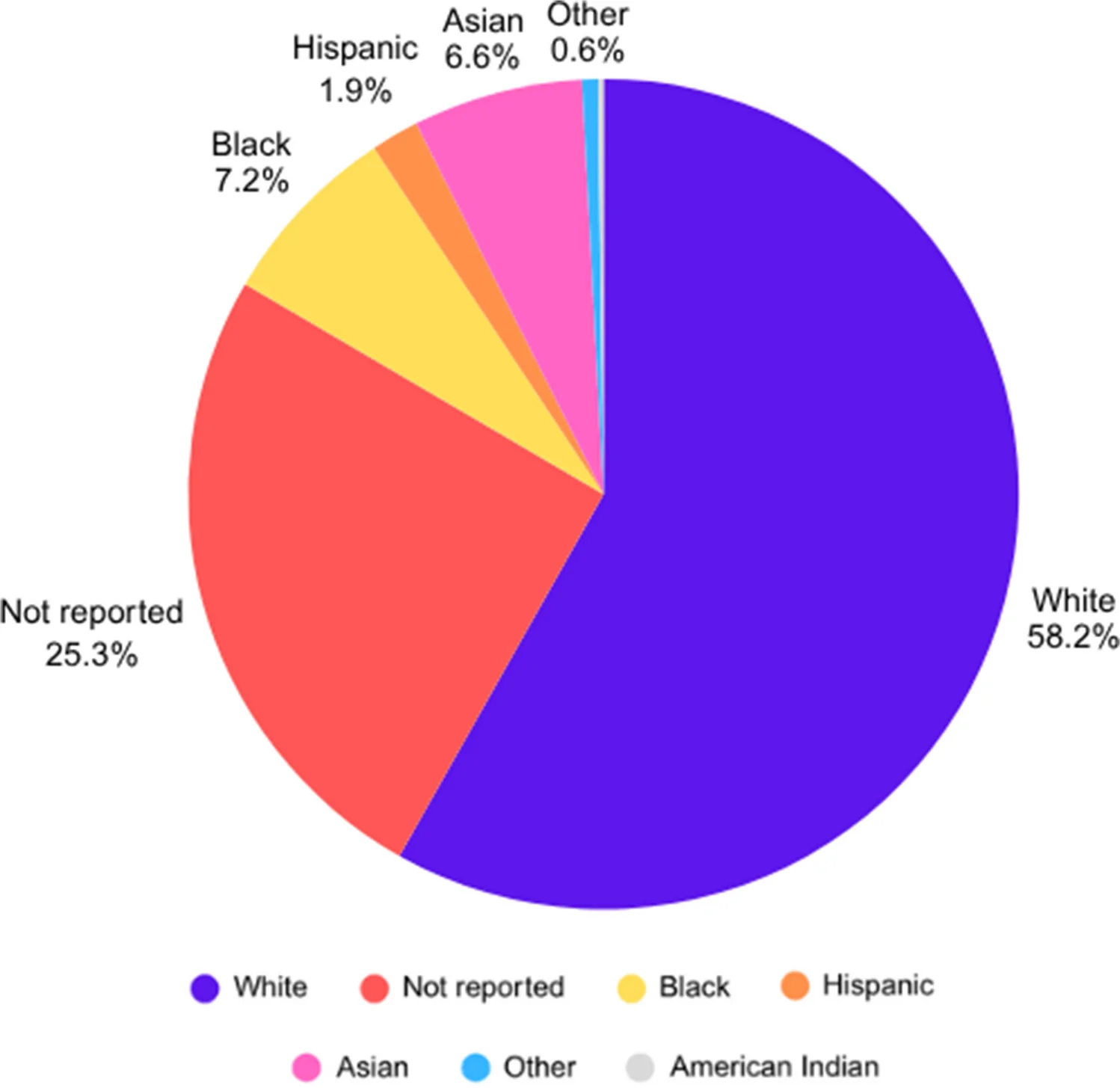
A pie chart showing the ethnic background of participants across all included studies

Figure 3 presents ethnic background reported across studies. There was limited diversity within the studies in terms of ethnicity, with 7% identifying as Black (n = 35) and 6% as Asian (n = 32). The mean age was 60.3 years (range = 53 to 64.8 years old). Three interventions engaged underrepresented groups through cultural adaptation: a lifestyle intervention adapted for Chinese women living in Hong Kong; a physical activity intervention for socio-culturally diverse women with obesity living in the Bronx, New York; and a lifestyle intervention for African American women in the USA [47, 49, 50]. Intervention tailoring strategies are included in Table 1.

### Intervention characteristics

Twelve interventions were described across 22 articles (Table 1). Half (n = 6) targeted physical activity only: 4 telephone-based, 1 supervised exercise at a gym, and 1 combining supervised exercise and home-based walking. Activities included aerobic, strength training, and moderate-intensity walking. Intervention duration ranged from 10 weeks to 6 months. Most were supported by educational materials, telephone coaching, or group counselling.

Combined diet and exercise interventions accounted for 25% (n = 3). These interventions targeted dietary habits and physical activity levels for improving HRQoL, cardiorespiratory fitness, or achieving weight loss. Delivery formats were generally group-based and face-to-face, incorporating behavioural counselling to enhance self-efficacy and promote sustainable lifestyle changes. The remaining 25% of interventions (n = 3) promoted broader lifestyle change, integrating diet, physical activity, and an additional component such as mindfulness, exercise self-efficacy, or health education. Education topics included health screening attendance, chronic disease prevention, and menopause symptom management. Intervention duration ranged from 12 to 14 weeks; delivery was online or a hybrid format of face-to-face group exercise with home-based activity targets.

### Results from randomised controlled trials

One powered RCT (“SUCCEED”) evaluated a physical activity and diet intervention [51]. The primary outcome was weight change at 12 months (–4.6 kg in the intervention group). Secondary outcomes included physical activity (Godin Leisure-Time Exercise questionnaire), dietary intake, and waist circumference. Physical activity minutes were calculated as the sum of moderate-intensity minutes plus twice the number of vigorous-intensity minutes. Significant increases in minutes of physical activity were reported in the intervention group (mean difference of 100 min at 6 months and 89 min at 12 months). Adherence was high (84%), and attrition was lower (14.6%, n = 6) in the intervention group compared to controls (29.4%, n = 10). The participants were predominantly of White ethnicity (91%).

### Results from pilot and feasibility trials

Nine pilot RCTs and feasibility trials assessed the feasibility, acceptability, and preliminary efficacy of interventions. There was variability across studies in the way physical activity was operationalised and assessed. Among the 12 interventions, 4 used only self-reported measures of physical activity (using the Stanford 7-Day Physical Activity Recall, Godin Leisure-Time Exercise questionnaire, or the modified Paffenbarger Harvard Alumni Activity Survey); 5 studies employed a combination of self-report and objective measures of physical activity (often accelerometry or FitBit data with questionnaires, ecological momentary assessment, or exercise logs). Three studies did not measure physical activity, instead monitoring intervention adherence, anthropometry, exercise self-efficacy, or physical function. Quality of life (QoL) was a secondary outcome measure in 10 trials (83%).

Five interventions achieved high adherence rates (75–86%) [52–56]. Three reported “moderate”, “satisfactory” or “reasonable” adherence, though these terms were not clearly defined or quantified [49, 57, 58]. One study reported low adherence (36%) [59]. Studies reporting high adherence varied considerably in terms of intervention design and delivery, though all 5 involved structured, weekly sessions across 8 to 12 weeks. Two focused solely on physical activity. “EPEC-FAST” provided a 10-week, personalised exercise programme through one-to-one sessions with a personal trainer, which was feasible and showed preliminary improvements in QoL, physical fitness, and weight loss [54]. This was assessed using the EORTC QLQ-C30, six-minute walk test, and anthropometry. Gorzelitz offered a 10-week, home-based exercise programme, and reported this to be feasible (primary outcome; examined adherence, satisfaction, and safety) with improvements observed in some tests of the Functional Fitness Battery but not all [60].

Two interventions integrated physical activity and diet components. “Shape-Up Following Cancer Treatment”, an 8-week, group programme underpinned by social cognitive theory, improved diet and quality of life, but not physical activity or weight (assessed using the AHEI-2010, EORTC-QLQ-C30, and Stanford 7-Day Physical Activity Recall) [13]. Schwartz delivered a 12-week walking and diet intervention, comprising 5 weekly walking sessions, a 10,000 daily steps target, and dietician support [53]. The intervention was deemed feasible (primary outcome; examined adherence to walking sessions using Fitbit data and exercise diary) despite a 47% participant withdrawal. Improvements were noted in body composition and immune response. The final intervention reporting high adherence (“MIM + D”) consisted of 8 weeks of group-based mindful yoga and diet sessions followed by 5 weeks of home-based practice [55]. Feasibility was demonstrated (primary outcome; adherence and satisfaction) but no changes in physical activity, physical function, or QoL (modified Paffenbarger Physical Activity Questionnaire, accelerometry, Short Physical Performance Battery, RAND SF-36).

In pilot and feasibility trials reporting moderate to low adherence, 1 lifestyle intervention combined diet, physical activity, and health promotion over 12 weeks via a website [49]. Including 54% endometrial cancer participants, feasibility was confirmed (primary outcome; recruitment, retention, safety, and engagement), with small gains in exercise self-efficacy and quality of life (secondary outcomes; FACT-G, Hospital Anxiety and Depression Scale, Eating Habits Self-efficacy Scale, Exercise Self-efficacy Scale). Rahimy et al. evaluated a 5-month Fitbit walking programme, with step goals and remote counselling [58]. No change in step count at the 9-month follow-up (primary outcome) or BMI was observed. Another intervention was a 6-month exercise programme for women with endometrial cancer (58% of participants) and obesity [59]. It constituted daily walking, strength training, and telephone coaching. Positive changes in quality of life and step count were observed in 36% of the sample completing the intervention (primary outcomes; FACT-G, exercise logs). Factors contributing to lower adherence included lower baseline mental QOL or activity levels. Intervention timing was important, with feedback from participants over 1 year post-treatment recommending interventions be initiated earlier in the survivorship period [55]. Electronic support formats were also discussed in relation to low adherence; one study reported no increase in step count from baseline in the group receiving electronic support compared to telephone support, another study reported that an online discussion forum was not used by participants [49, 58].

### Results from qualitative data

Three studies conducted an embedded qualitative study to explore participant perceptions, motivations, barriers, and perceived benefits. One study was embedded within a pilot feasibility RCT of a culturally tailored physical activity intervention for socio-culturally diverse, obese women treated for endometrial cancer [46]. Semi-structured interviews with 16 intervention completers were conducted, though participant characteristics were not reported alongside qualitative findings. Thematic analysis identified key motivators including health improvement, maintaining independence, and weight loss. Social connectedness and group support were important facilitators of adherence, while fatigue, weather, pain, and caregiving responsibilities were common barriers. Participants reported enhanced self-efficacy, physical functioning, body composition, and improved ability to perform activities of daily living. Another study embedded within a pilot RCT involved semi-structured interviews with completers of a culturally adapted lifestyle intervention in Hong Kong (n = 13) [49]. The sample consisted of 53.8% endometrial cancer from a range of socioeconomic backgrounds (5 low-income, 6 middle-income, and 4 high-income). Ethnic background was not explicitly reported. Key themes included perceived benefits and impressions of the intervention, supported by subthemes of useful health-promoting information, new knowledge, positive lifestyle changes, and coping better with adverse treatment effects. One protocol for a feasibility trial included plans for a qualitative evaluation, but qualitative data were not included in the subsequent results paper and have not yet been published [54, 61].

### Use of behaviour change theory

Five of the 12 interventions reported using a behaviour change theory to inform their design [47, 48, 50, 62, 63]. Social Cognitive Theory (SCT) was utilised in all cases, either alone (n = 3) or in combination with another framework (n = 2). SCT considers self-efficacy to be the central mechanism influencing behaviour change, with outcome expectations, goals, knowledge, and perceived barriers and facilitators as key determinants [64]. Among the theory-based interventions included in this review, SCT was operationalised to increase self-efficacy through strategies including goal setting, self-monitoring, self-regulation, problem solving, and the use of group settings to facilitate observational learning and social support. One intervention additionally incorporated Control Theory, using behavioural feedback (participants received feedback on their progress toward goals) and adjustment (behavioural modification was encouraged if goals were not met) [62]. Another intervention combined SCT with the Health Belief Model to target perceived benefits of desired behaviour, perceived barriers, and cues to action [47].

Outcome data were reported for 3 of the 5 theory-informed interventions. The “SUCCEED” intervention, underpinned by SCT, significantly increased physical activity minutes and step count in an RCT [63]. Another SCT-based intervention improved self-efficacy and physical function in a feasibility trial; however increases in self-reported physical activity were not maintained over time [50]. The intervention combining SCT and Control Theory improved diet and quality of life but did not increase physical activity in a pilot trial [62].

### Considerations for future research

Authors of included studies identified several priorities for future research on physical activity interventions in women treated for endometrial cancer. Multiple studies emphasised the importance of tailoring interventions to participants’ baseline characteristics; particularly BMI, mental health status, and baseline activity level [54, 59, 65]. Intervention tailoring based on participant response was also proposed, such as increasing exercise difficulty for highly engaged participants and providing additional or longer-term support for less responsive participants [54, 60]. Higher baseline mental QoL was associated with trial completion and intervention benefit in one trial; authors suggested that supporting psychological wellbeing prior to intervention delivery may facilitate engagement [59]. Adherence and engagement may also be improved by integrating interventions earlier in survivorship, adapting study demands to participants’ quality of life, or considering entry cut-offs for HRQoL [55, 56, 58, 66].

Two studies advised using objective physical activity measures, such as activity trackers supplemented with logbooks, to minimise bias from self-reporting [58, 62]. Stratifying analyses by cancer stage and treatment history to identify potential effect modifiers was also suggested [67].

Several studies emphasised the need for greater inclusion of socio-culturally diverse participants in future trials. One feasibility study specifically targeting underserved populations reported challenges recruiting through oncology clinics and support groups, but found that once enrolled, attendance to exercise classes was high [50]. Authors recommended the use of theory-based interventions, incorporating peer mentors and social support, and addressing common forms of routine physical activity (e.g. walking, household chores) to support participation and maintain the benefits of physical activity interventions.

Collectively, authors called for further research to determine the optimal types, doses, and contexts of physical activity in this population. The consensus across all included studies was that the development of more effective interventions to support the adoption and maintenance of a physically active lifestyle will improve health and quality of life outcomes among women treated for endometrial cancer.

## Discussion

### Summary of key findings

This scoping review assessed the extent, range, and nature of existing physical activity interventions for women treated for endometrial cancer. A total of 12 interventions were identified across 22 studies, with most evidence derived from feasibility or pilot trials. Only one powered RCT was identified, reflecting the early stage of intervention development in this field and suggesting a likely progression toward larger, definitive trials. Feasibility was consistently demonstrated, with high adherence reported in 5 studies. Physical activity improvements were often modest, with increases noted in some studies but not consistent or sustained in others. The evidence base for physical activity interventions in women treated for endometrial cancer is predominantly generated in high-income Western contexts. The ethnic background of participants sampled across all studies was primarily White, highlighting the need for more diverse and representative samples in future research.

Less than half of the interventions reported using behaviour change theory, suggesting that existing interventions may not optimally target the mechanisms that underpin sustained behaviour change. Of those that did, only social cognitive theory-informed interventions have been evaluated, with mixed findings, particularly for the maintenance of physical activity over longer follow-up periods. The predominance of a single theoretical approach indicates the potential value of more comprehensive frameworks, such as the Behaviour Change Wheel, which integrates constructs from SCT and other behavioural theories [68]. Only one study included in this review examined whether changes in physical activity were mediated by proposed mechanisms, emphasising the need for future research to test both intervention efficacy and underlying mechanisms of action [48]. The relevance of these mechanisms of action may differ between the initiation and maintenance of physical activity. Another key gap is the absence of co-design across all included studies. None of the interventions reported involving women with lived experience, healthcare professionals, or public contributors in its development. Co-designed interventions have the potential to better align with women’s priorities and lived experiences, particularly in populations where healthcare access, stigma, and financial constraints may shape engagement with lifestyle change.

A strength across the evidence base is the growing use of remote and home-based delivery formats, which have the potential to enhance reach and scalability. Several interventions were telephone-delivered, online, or incorporated home-based physical activity ‘prescriptions’, and showed promise in improving outcomes for women managing cancer alongside obesity [58]. However longer-term adherence remains underexplored, and electronic support (websites, online forums, electronic reminders) was not well adhered to [49]. In contrast, some interventions relied on research-controlled or supervised hospital settings, offering greater structure but may not reflect real-world conditions and could limit long-term adherence, especially as only a few incorporated follow-up support.

A challenge in this area is the lack of standardised physical activity measurement across studies. Earlier studies relied primarily on self-report instruments, whereas studies conducted from 2016 onward have increasingly adopted objective measures such as accelerometers and other wearable devices. This shift reflects the growing accessibility and acceptability of digital technologies among women treated for endometrial cancer [69]. To strengthen methodological rigour and comparability across studies, future research should use validated objective measures of physical activity and draw on emerging best-practice protocols for accelerometer data collection and quality assurance [70].

Almost all studies included quality of life as a secondary outcome measure; often using a combination of general health-related quality of life and endometrial-cancer-specific instruments. FACT-G and FACT-En have been used with increasing consistency across included studies, with SF-36 variants representing the next most commonly used measures of QoL. While generic measures (FACT-G, SF-36) facilitate cross-condition comparisons and are frequently selected to support health economic evaluation, disease-specific QoL instruments (FACT-En, EORTC QLQ-EN24) are more sensitive to the physical, psychological, and functional impacts of endometrial cancer and its treatment. The consistent use of FACT-G and FACT-En as validated, cancer-specific instruments would improve the comparability of QoL measurement across trials and contribute to future evidence synthesis of both endometrial cancer-specific and cross-cancer populations. Together, these tools reflect an evolving understanding of survivorship in which wellbeing is increasingly recognised as a core outcome. Improvements in health-related QoL were consistently observed across studies despite small or inconsistent changes in physical activity behaviour, suggesting that even modest increases in physical activity may bring about QoL benefits that are important to women treated for endometrial cancer. However, comparing QoL outcomes across studies is challenging, in part due to heterogeneity in outcome measures and lack of consensus around thresholds for clinically meaningful change within endometrial cancer [71]. A more detailed understanding of which domains are most relevant to women treated for endometrial cancer and how they are influenced by physical activity may also help strengthen outcome selection and inform the design of future interventions.

### Implications and directions for future research

There is a growing recognition of the importance of addressing both physical and psychological well-being in the context of lifestyle change interventions, as evidenced by the integration of mindfulness into one diet and physical activity intervention, and another study reporting that baseline mental quality of life predicted better outcomes and intervention adherence [55, 59]. These findings suggest that participants may require different levels of input based on their readiness, fitness, or psychological well-being. Tailoring strategies such as entry screening, stepped-intensity models, or the integration of psychological support could enhance adherence and acceptability. More broadly, these findings underscore the importance of equity-informed intervention design that acknowledges differential starting points in terms of participants’ capacity or needs and avoids a one-size-fits-all approach.

Cultural relevance also warrants greater attention. Three interventions were tailored to women from African American and Chinese ethnic backgrounds [47, 49, 50], yet the extent of cultural tailoring was inconsistent and only one study reported using a cultural adaptation framework [49]. Future work should build on this progress by embedding cultural tailoring systematically, through content adaptation and the inclusion of representatives from different backgrounds as collaborators and patient and public advisors in intervention development. Further progress towards culturally tailored interventions is vital for ensuring that interventions are generalisable, acceptable, and effective across different cultural contexts.

Overall, the value and potential of behavioural lifestyle programs for EC patients was recognised across all studies. Long-term adherence remains insufficiently understood. Future research should further examine the behavioural mechanisms through which physical activity interventions may exert their effects. Some included studies reported improvements in quality of life, self-efficacy, or physical functioning even in the absence of substantial increases in physical activity levels. This suggests that changes in behavioural determinants such as motivation or confidence may play a role in driving observed benefits. Testing these mechanisms explicitly whilst accounting for baseline mental QoL, physical activity levels, and obesity status in future trials may help clarify how interventions work and for whom.

### Strengths and limitations

The use of a systematic scoping review framework supports transparency and replicability [40]. Consistent with this approach, no quality appraisal or risk of bias assessment was conducted, which limits the ability to comment on the internal validity or reliability of included studies. The absence of patient and public involvement in developing the review question or interpreting the findings may also limit the relevance and applicability of the conclusions to women treated for endometrial cancer. A strength of the review is the broad inclusion criteria, which encompassed grey literature, trial protocols, and qualitative studies alongside trials reporting primary outcomes. This enabled a comprehensive synthesis of physical activity interventions for women treated for endometrial cancer, capturing early-stage intervention development, implementation processes, and participant experiences. The review was limited to women who had completed primary treatment for endometrial cancer, excluding those undergoing active treatment. This decision reflects the post-active treatment phase as a distinct and underexplored period of survivorship, in which individuals rebuild routines following clinical discharge and may be receptive to making lifestyle changes as a form of control over the risk of cancer recurrence [72]. Interventions delivered during active treatment often serve different purposes, typically aiming to optimise treatment effectiveness, mitigate acute toxicity, or preserve function. Making this distinction enabled a focused synthesis of interventions designed to support long-term physical activity behaviour change after treatment, but future work may benefit from exploring the effectiveness of physical activity interventions in patients across these phases to inform the potential for physical activity support spanning diagnosis to recovery.

## Conclusions

This scoping review highlights promising but preliminary evidence for the feasibility and benefits of physical activity interventions for women treated for endometrial cancer. The predominance of pilot and feasibility studies in this area indicates a need for progression toward larger, definitive trials. To advance the field, three priorities emerge. First, the involvement of women with lived experience of endometrial cancer—through co-design and patient and public involvement—is needed to ensure interventions are aligned with service user needs. Secondly, greater attention to cultural tailoring and equity-informed intervention design is essential to improve uptake among underrepresented populations. Finally, future trials should consider intervention tailoring to account for factors shown to affect adherence, such as physical activity levels, the presence of comorbid conditions including obesity, and mental quality of life at the point of study entry.

## Supplementary Information

Below is the link to the electronic supplementary material.

Supplementary file1 (DOCX 39 KB)

## Data Availability

No datasets were generated or analysed during the current study.
