# Supplementary material for: Physical activity interventions in women treated for endometrial cancer: a scoping review
Source: J Cancer Surviv. 2025 Nov 28;20(5):2807–24. doi: 10.1007/s11764-025-01938-7 (PMC13723445; doi:10.1007/s11764-025-01938-7)
Supplement: Supplementary file 1 [file 11764_2025_1938_MOESM1_ESM.docx]

**Appendix 1 | SEARCH STRATEGY**

**Medline**

Ovid MEDLINE(R) ALL <1946 to October 23, 2023>

1	exp Uterine Neoplasms/

2	((uterus or uterine or endometri* or womb or corpus uteri) adj5 (cancer* or tumor* or tumour* or neoplas* or carcinoma* or adenocarcinoma* or malignan*)).tw,kf.

3	1 or 2

4	exp Exercise/

5	exp sports/

6	dancing/

7	exp Exercise Therapy/

8	weight-lifting/

9	high intensity interval training/

10	Rehabilitation/

11	(physical adj3 activit*).tw,kf.

12	(exercise or exercises or exercising).tw,kf.

13	(physical adj3 (therap* or train* or conditioning)).tw.

14	(running or jogging).tw,kf.

15	swim*.tw,kf.

16	walk*.tw,kf.

17	(dance or dancing).tw,kf.

18	sport*.tw,kf.

19	(bike* or bicycl*).tw,kf.

20	gym*.tw,kf.

21	resistance training.tw,kf.

22	strengthening program*.tw,kf.

23	weight-lifting.tw,kf.

24	interval training.tw,kf.

25	(prehabilitation or pre-habilitation or rehabilitation).tw,kf.

26	or/4-25 [Exercise]

27	exp animals/ not exp humans/

28	Comment/

29	Letter/

30	Editorial/

31	"Comment on".ti.

32	(letter* adj3 editor*).ti.

33	opinion*.ti.

34	28 or 29 or 30 or 31 or 32 or 33

35	3 and 26 [Endometrial Cancer and Exercise]

36	35 not 27 [Endometrial Cancer and Exercise_Animal studies removed]

37	36 not 34 [Endometrial Cancer and Exercise_Animal studies plus opinion articles removed]	1062

38	limit 37 to yr="2011 -Current"

**Embase**

Embase ALL <1946 to 2023 Week 37>

1	exp Uterine Neoplasms/	209414

2	((uterus or uterine or endometri* or womb or corpus uteri) adj5 (cancer* or tumor* or tumour* or neoplas* or carcinoma* or adenocarcinoma* or malignan*)).tw,kf.	79686

3	1 or 2	224434

4	exp Exercise/	382589

5	exp sports/	187314

6	dancing/	5984

7	exp Exercise Therapy/	90776

8	weight-lifting/	4877

9	high intensity interval training/	4583

10	Rehabilitation/	76604

11	(physical adj3 activit*).tw,kf.	207486

12	(exercise or exercises or exercising).tw,kf.	413319

13	(physical adj3 (therap* or train* or conditioning)).tw.	55688

14	(running or jogging).tw,kf.	79423

15	swim*.tw,kf.	49806

16	walk*.tw,kf.	193085

17	(dance or dancing).tw,kf.	9091

18	sport*.tw,kf.	125022

19	(bike* or bicycl*).tw,kf.	31156

20	gym*.tw,kf.	13627

21	resistance training.tw,kf.	13772

22	strengthening program*.tw,kf.	693

23	weight-lifting.tw,kf.	1095

24	interval training.tw,kf.	5559

25	(prehabilitation or pre-habilitation or rehabilitation).tw,kf.	266085

26	or/4-25 [Exercise]	1302051

27	exp animals/ not exp humans/	3542110

28	Comment/	1

29	Letter/	940681

30	Editorial/	622568

31	"Comment on".ti.	29473

32	(letter* adj3 editor*).ti.	34816

33	opinion*.ti.	18101

34	28 or 29 or 30 or 31 or 32 or 33	1599346

35	3 and 26 [Endometrial Cancer and Exercise]	2778

36	35 not 27 [Endometrial Cancer and Exercise_Animal studies removed]	2757

37	36 not 34 [Endometrial Cancer and Exercise_Animal studies plus opinion articles removed]	2695

38	limit 37 to yr="2011 -Current"	1989

1     [result of topic search].

2     exp animals/ not exp human/

3     exp nonhuman/ not exp human/

4     exp experimental animal/

5     exp veterinary medicine/

6     animal experiment/

7     or/2-6

8     1 NOT 7

**Cochrane Library**

1	MeSH descriptor: [Uterine Neoplasms] explode all trees

2	(uterus OR uterine OR endometrial OR womb OR "corpus uteri"):ti,ab,kw

3	((cancer) OR (tumour) OR (neoplasm) OR (carcinoma) OR (malignancy)):ti,ab,k

4	#2 AND #3

5	#1 OR #4

6	(“high intensity interval training”):ti,ab,kw

7	("rehabilitation"):ti,ab,kw

8	(physical NEAR/3 activit*):ti,ab,kw

9	(exercise OR exercises OR exercising):ti,ab,kw

10	(physical NEAR/3 (therap* OR train* OR conditioning)):ti,ab,kw

11	(running OR jogging):ti,ab,kw

12	(swim*):ti,ab,kw

13	(walk*):ti,ab,kw

14	(dance OR dancing):ti,ab,kw

15	(sport):ti,ab,kw

16	((bike*) OR (bicycl*)):ti,ab,kw

17	(gym*):ti,ab,kw

18	(sports):ti,ab,kw

19	("weight lifting"):ti,ab,kw

20	("weight training"):ti,ab,kw

21	(“resistance training”):ti,ab,kw

22	(strengthening NEXT program*):ti,ab,kw

23	(“interval training”):ti,ab,kw

24	(prehabilitation):ti,ab,kw

25	#6 OR #7 OR #8 OR #9 OR #10 OR #11 OR #12 OR #13 OR #14 OR #15 OR #16 OR #17 OR #18 OR #19 OR #20 OR #21 OR #22 OR #23 OR #24 [Exercise]

26	rat:ti OR rats:ti OR mouse:ti OR mice:ti OR swine:ti OR porcine:ti OR murine:ti OR sheep:ti OR lambs:ti OR pigs:ti OR piglets:ti OR rabbit:ti OR rabbits:ti OR cat:ti OR cats:ti OR dog:ti OR dogs:ti OR cattle:ti OR bovine:ti OR monkey:ti OR monkeys:ti OR trout:ti OR marmoset$:ti

27	#5 AND #25 [Endometrial Cancer and Exercise]

28	#27 NOT #26	[Endometrial Cancer and Exercise_Animal studies removed]

29	#28 with Cochrane Library publication date from Jan 2011 to Oct 2023

**Web of Science**

Web of Science Core Collection ALL

1	ALL=(Uterine Neoplasm)

2	TS=((uterus OR uterine OR endometri* OR womb OR "corpus uteri") NEAR/5 (cancer* OR tumor* OR tumour* OR neoplas* OR carcinoma* OR adenocarcinoma* OR malignan*))

3	TS=(1 or 2)

4	(TS=("exercise" OR "physical activity"))

5	TS=("sports" OR "athletics" OR "athletic" OR "sporting")

6	TS=(dancing)

7	TS=("Exercise Therapy" OR "Exercise-Based Therapy" OR "Therapeutic Exercise")

8	TS=(weight-lifting)

9	TS=(high intensity interval training)

10	TS=(Rehabilitation)

11	TS=(physical NEAR/3 activit*)

12	TS=((exercise or exercises or exercising))

13	TS=(("physical" NEAR/3 (therap* OR train* OR conditioning)))

14	TS=("running" OR "jogging")

15	TS=(swim*)

16	TS=(walk*)

17	TS=(dance or dancing)

18	TS=(sport*)

19	TS=(bike* or bicycl*)

20	TS=(gym*)

21	TS=(resistance training)

22	TS=(strengthening program*)

23	TS=(weight-lifting)

24	TS=(interval training)

25	TS=((prehabilitation or pre-habilitation or rehabilitation))

26	#4 OR #5 OR #6 OR #7 OR #8 OR #9 OR #10 OR #11 OR #12 OR #13 OR #14 OR #15 OR #16 OR #17 OR #18 OR #19 OR #20 OR #21 OR #22 OR #23 OR #24 OR #25

27	ALL=(comment)

28	ALL=(letter)

29	ALL=(editorial)

30	TS=(Comment on)

31	TI=(letter* NEAR/3 editor*)

32	TI=(opinion*)

33	#27 OR #28 OR #29 OR #30 OR #31 OR #32

34	#3 AND #26 [Endometrial Cancer and Exercise]

35	TS=(Animals NOT Humans)

36	#34 NOT #35 [Endometrial Cancer and Exercise_Animal studies removed]

37	#34 not #32 [Endometrial Cancer and Exercise_Animal studies plus opinion articles removed]

38	PY=(2011-2023)

39	#37 AND #38 [Endometrial Cancer and Exercise_Animal studies_opinion articles removed plus published 2011-2023]

40	#3 AND #33 AND #34 AND #36 AND #37 AND #39 AND #26

**PsychInfo**

APA PsycInfo ALL <2002 to October Week 3 2023>

1	Uterine Neoplasms.mp

2	((uterus or uterine or endometri* or womb or corpus uteri) adj5 (cancer* or tumor* or tumour* or neoplas* or carcinoma* or adenocarcinoma* or malignan*)).tw,id.

3	1 or 2

4	exp Exercise/

5	exp sports/

6	dancing/

7	exp Exercise Therapy/

8	weight-lifting/

9	high intensity interval training/

10	Rehabilitation/

11	(physical adj3 activit*).tw,id.

12	(exercise or exercises or exercising).tw,id.

13	(physical adj3 (therap* or train* or conditioning)).tw.

14	(running or jogging).tw,id.

15	swim*.tw,id.

16	walk*.tw,id.

17	(dance or dancing).tw,id.

18	sport*.tw,id.

19	(bike* or bicycl*).tw,id.

20	gym*.tw,id.

21	resistance training.tw,id.

22	strengthening program*.tw,id.

23	weight-lifting.tw,id.

24	interval training.tw,id.

25	(prehabilitation or pre-habilitation or rehabilitation).tw,id.

26	or/4-25 [Exercise]

27	(animal not (animal and human)).po.

28	comment reply.dt.

29	letter.dt.

30	editorial.dt.

31	"Comment on".ti.

32	(letter* adj3 editor*).ti.

33	opinion*.ti.

34	"review book".dt.

35	28 or 29 or 30 or 31 or 32 or 33 or 34

36	3 and 26 [Endometrial Cancer and Exercise]

37	35 not 27 [Endometrial Cancer and Exercise_Animal studies removed]

38	36 not 34 [Endometrial Cancer and Exercise_Animal studies plus opinion articles removed]

39	limit 37 to yr="2011 -Current"

**PEDro**

PEDro

Search 1:

Abstract & Title: uterine

Therapy:

Problem: [no appropriate value in this field]

Body part:

Subdiscipline: oncology

Published since: 2011

Match all search terms (AND)

Results:

| Title | Method | Score (/10) |
| --- | --- | --- |
| [The use of acupuncture for managing gynaecologic conditions: an overview of systematic reviews](https://www.sciencedirect.com/science/article/pii/S0378512211000363) | Systematic review | N/A |
| [Survivors of uterine cancer empowered by exercise and healthy diet (SUCCEED): a randomized controlled trial](https://pubmed.ncbi.nlm.nih.gov/22465522/) | Clinical trial | 6/10 |
| [Self-efficacy, quality of life, and weight loss in overweight/obese endometrial cancer survivors (SUCCEED): a randomized controlled trial](https://www.sciencedirect.com/science/article/pii/S0090825813013875) | Clinical trial | 3/10 |

Search 2:

Abstract & Title: womb

Therapy: strength training

Problem:

Body part:

Subdiscipline: oncology

Published since: 2011

Match all search terms (AND)

No records found

Search 3:

Abstract & Title: womb

Therapy: fitness training

Problem:

Body part:

Subdiscipline: oncology

Published since: 2011

Match all search terms (AND)

No records found

Search 4:

Abstract & Title: womb

Therapy: behaviour modification

Problem:

Body part:

Subdiscipline: oncology

Published since: 2011

Match all search terms (AND)

No records found

Search 5:

Abstract & Title: exercis* "physical activity"

Therapy:

Problem:

Body part:

Subdiscipline: oncology

Published since: 2011

Match all search terms (AND)

Results: 389

All 389 search results were screened based on titles. Any reference to a specific cancer group unrelated to endometrial cancer was omitted from the results (prostate, colon, breast, metastatic breast, lung) or references to treatments not applicable to endometrial cancer (mastectomy, radical prostatectomy, robotic prostatectomy) were also omitted. 73 results remained for title & abstract screening:

| # | Title | PEDro link | Full text link |
| --- | --- | --- | --- |
| 1 | (Physical activity and exercise recommendations for cancer patients during rehabilitation) [German] | [Record](https://search.pedro.org.au/search-results/record-detail/41464) | [Full text](https://www.thieme-connect.de/products/ejournals/abstract/10.1055/s-0033-1334916) |
| 2 | Exercise interventions for adults with cancer receiving radiation therapy alone [with consumer summary] | [Record](https://search.pedro.org.au/search-results/record-detail/73071) | [Full text](https://www.cochranelibrary.com/cdsr/doi/10.1002/14651858.CD013448.pub2/full) |
| 3 | Interventions for promoting habitual exercise in people living with and beyond cancer (Cochrane review) [with consumer summary] | [Record](https://search.pedro.org.au/search-results/record-detail/36969) | [Full text](https://www.cochranelibrary.com/cdsr/doi/10.1002/14651858.CD010192.pub3/full) |
| 4 | Exercise for the management of cancer-related fatigue in adults (Cochrane review) [with consumer summary] | [Record](https://search.pedro.org.au/search-results/record-detail/20432) | [Full text](https://www.cochranelibrary.com/cdsr/doi/10.1002/14651858.CD006145.pub3/full) |
| 5 | Social media interventions targeting exercise and diet behaviours in people with noncommunicable diseases (NCDs): a systematic review [with consumer summary] | [Record](https://search.pedro.org.au/search-results/record-detail/70092) | [Full text](https://www.sciencedirect.com/science/article/pii/S2214782922000045?via%3Dihub) |
| 6 | (Physical activity in cancer-effectiveness of exercise therapy approaches) [German] | [Record](https://search.pedro.org.au/search-results/record-detail/71496) | [Full text](https://www.thieme-connect.de/products/ejournals/abstract/10.1055/a-1685-6919) |
| 7 | Effectiveness of physical activity on immunity markers and quality of life in cancer patient: a systematic review | [Record](https://search.pedro.org.au/search-results/record-detail/71905) | [Full text](https://peerj.com/articles/13664/) |
| 8 | Home-based aerobic and resistance exercise interventions in cancer patients and survivors: a systematic review [with consumer summary] | [Record](https://search.pedro.org.au/search-results/record-detail/64544) | [Full text](https://www.mdpi.com/2072-6694/13/8/1915) |
| 9 | Effectiveness of smartphone-based physical activity interventions on individuals' health outcomes: a systematic review | [Record](https://search.pedro.org.au/search-results/record-detail/66669) | [Full text](https://www.hindawi.com/journals/bmri/2021/6296896/) |
| 10 | The effect of exercise-based interventions on health-related quality of life and physical function in older patients with cancer receiving medical antineoplastic treatments: a systematic review | [Record](https://search.pedro.org.au/search-results/record-detail/62868) | [Full text](https://eurapa.biomedcentral.com/articles/10.1186/s11556-020-00250-w) |
| 11 | Group-based exercise interventions for increasing physical activity in cancer survivors: a systematic review of face-to-face randomized and non-randomized trials | [Record](https://search.pedro.org.au/search-results/record-detail/57341) | [Full text](https://link.springer.com/article/10.1007/s00520-019-04670-y) |
| 12 | Exercise on quality of life and cancer-related fatigue for lymphoma survivors: a systematic review and meta-analysis | [Record](https://search.pedro.org.au/search-results/record-detail/57891) | [Full text](https://link.springer.com/article/10.1007/s00520-019-04983-y) |
| 13 | The effects of physical activity on cancer prevention, treatment and prognosis: a review of the literature [with consumer summary] | [Record](https://search.pedro.org.au/search-results/record-detail/59593) | [Full text](https://www.sciencedirect.com/science/article/pii/S0965229918309646?via%3Dihub) |
| 14 | Exercise and other non-pharmaceutical interventions for cancer-related fatigue in patients during or after cancer treatment: a systematic review incorporating an indirect-comparisons meta-analysis [with consumer summary] | [Record](https://search.pedro.org.au/search-results/record-detail/49745) | [Full text](https://bjsm.bmj.com/content/52/10/651) |
| 15 | Biological markers as an outcome measure of exercise in cancer rehabilitation: a systematic review | [Record](https://search.pedro.org.au/search-results/record-detail/52713) | [Full text](https://journals.lww.com/cancerjournal/fulltext/2018/14020/biological_markers_as_an_outcome_measure_of.5.aspx) |
| 16 | The feasibility, acceptability and outcomes of exergaming among individuals with cancer: a systematic review | [Record](https://search.pedro.org.au/search-results/record-detail/55571) | [Full text](https://bmccancer.biomedcentral.com/articles/10.1186/s12885-018-5068-0) |
| 17 | The role of physical activity in managing fatigue in cancer survivors | [Record](https://search.pedro.org.au/search-results/record-detail/58151) | [Full text](https://link.springer.com/article/10.1007/s13668-018-0234-1) |
| 18 | Does psychoeducation added to oncology rehabilitation improve physical activity and other health outcomes? A systematic review | [Record](https://search.pedro.org.au/search-results/record-detail/49985) | [Full text](https://journals.lww.com/rehabonc/abstract/2017/04000/does_psychoeducation_added_to_oncology.5.aspx) |
| 19 | Effects of exercise interventions and physical activity behavior on cancer related cognitive impairments: a systematic review | [Record](https://search.pedro.org.au/search-results/record-detail/45801) | [Full text](https://www.hindawi.com/journals/bmri/2016/1820954/) |
| 20 | Exercise interventions for patients with gynaecological cancer: a systematic review and meta-analysis | [Record](https://search.pedro.org.au/search-results/record-detail/48308) | [Full text](https://www.physiotherapyjournal.com/article/S0031-9406(16)30001-3/fulltext) |
| 21 | Addition of motivational interventions to exercise and traditional physiotherapy: a review and meta-analysis | [Record](https://search.pedro.org.au/search-results/record-detail/42234) | [Full text](https://www.physiotherapyjournal.com/article/S0031-9406(14)00057-1/fulltext) |
| 22 | (Prehabilitation. Preparing patients for surgery to improve functional recovery and reduce postoperative morbidity) [French] | [Record](https://search.pedro.org.au/search-results/record-detail/38829) | [Full text](https://pubmed.ncbi.nlm.nih.gov/24440732/) |
| 23 | Predicting exercise adherence in cancer patients and survivors: a systematic review and meta-analysis of motivational and behavioural factors | [Record](https://search.pedro.org.au/search-results/record-detail/34489) | [Full text](https://onlinelibrary.wiley.com/doi/10.1111/j.1365-2702.2012.04322.x) |
| 24 | A systematic review of web-based interventions for patient empowerment and physical activity in chronic diseases: relevance for cancer survivors | [Record](https://search.pedro.org.au/search-results/record-detail/35155) | [Full text](https://www.jmir.org/2013/2/e37/) |
| 25 | Impact of resistance training in cancer survivors: a meta-analysis | [Record](https://search.pedro.org.au/search-results/record-detail/35696) | [Full text](https://journals.lww.com/acsm-msse/fulltext/2013/11000/impact_of_resistance_training_in_cancer_survivors_.8.aspx) |
| 26 | Physical activity and quality of life of cancer survivors: a lack of focus for lifestyle redesign | [Record](https://search.pedro.org.au/search-results/record-detail/36665) | [Full text](https://pubmed.ncbi.nlm.nih.gov/23725173/) |
| 27 | Physical activity for cancer survivors: meta-analysis of randomised controlled trials [with consumer summary] | [Record](https://search.pedro.org.au/search-results/record-detail/30886) | [Full text](https://www.bmj.com/content/344/bmj.e70) |
| 28 | Updated evidence in support of diet and exercise interventions in cancer survivors | [Record](https://search.pedro.org.au/search-results/record-detail/28017) | [Full text](https://www.tandfonline.com/doi/full/10.3109/0284186X.2010.529822) |
| 29 | (Constipation in cancer patients. Evidence for nursing interventions and promotion of physical activity) [Italian] | [Record](https://search.pedro.org.au/search-results/record-detail/31090) | [Abstract](https://pubmed.ncbi.nlm.nih.gov/22044542/) |
| 30 | Efficacy of health coaching and a web-based program on physical activity, weight, and distress management among cancer survivors: a multi-centered randomised controlled trial | [Record](https://search.pedro.org.au/search-results/record-detail/60759) | [Full text](https://onlinelibrary.wiley.com/doi/10.1002/pon.5394) |
| 31 | Effect of the LIVESTRONG at the YMCA exercise program on physical activity, fitness, quality of life, and fatigue in cancer survivors | [Record](https://search.pedro.org.au/search-results/record-detail/48829) | [Full text](https://acsjournals.onlinelibrary.wiley.com/doi/10.1002/cncr.30456) |
| 32 | A dance intervention for cancer survivors and their partners (RHYTHM) [with consumer summary] | [Record](https://search.pedro.org.au/search-results/record-detail/49951) | [Full text](https://link.springer.com/article/10.1007/s11764-016-0593-9) |
| 33 | Effect of a 12-month exercise intervention on serum biomarkers of angiogenesis in postmenopausal women: a randomized controlled trial | [Record](https://search.pedro.org.au/search-results/record-detail/39200) | [Full text](https://aacrjournals.org/cebp/article/23/4/648/70182/Effect-of-a-12-Month-Exercise-Intervention-on) |
| 34 | A patient education program is effective in reducing cancer-related fatigue: a multi-centre randomised two-group waiting-list controlled intervention trial | [Record](https://search.pedro.org.au/search-results/record-detail/35094) | [Full text](https://www.ejoncologynursing.com/article/S1462-3889(12)00068-3/fulltext) |
| 35 | Remotely delivered exercise to older rural cancer survivors: a randomized controlled pilot trial [with consumer summary] | [Record](https://search.pedro.org.au/search-results/record-detail/72608) | [Full text](https://link.springer.com/article/10.1007/s11764-022-01292-y) |
| 36 | Healthy moves to improve lifestyle behaviors of cancer survivors and their spouses: feasibility and preliminary results of intervention efficacy | [Record](https://search.pedro.org.au/search-results/record-detail/69464) | [Full text](https://www.mdpi.com/2072-6643/13/12/4460) |
| 37 | Examining the impact of an mHealth behavior change intervention with a brief in-person component for cancer survivors with overweight or obesity: randomized controlled trial | [Record](https://search.pedro.org.au/search-results/record-detail/71056) | [Full text](https://mhealth.jmir.org/2021/7/e24915) |
| 38 | (Structured evaluation of physical activity after cancer -- a randomized controlled trial) [German] | [Record](https://search.pedro.org.au/search-results/record-detail/61842) | [Abstract](https://www.dustri.com/article_response_page.html?artId=186765&doi=10.5414/PRX0547&L=1) |
| 39 | Diet, physical activity, and health-related outcomes of endometrial cancer survivors in a behavioral lifestyle program: the Diet and Exercise in Uterine Cancer Survivors (DEUS) parallel randomized controlled pilot trial | [Record](https://search.pedro.org.au/search-results/record-detail/56174) | [Full text](https://ijgc.bmj.com/content/29/3/531) |
| 40 | Feasibility, acceptability and efficacy of a text message-enhanced clinical exercise rehabilitation intervention for increasing 'whole-of-day' activity in people living with and beyond cancer | [Record](https://search.pedro.org.au/search-results/record-detail/58419) | [Full text](https://bmcpublichealth.biomedcentral.com/articles/10.1186/s12889-019-6767-4) |
| 41 | Cost-utility and cost-effectiveness of physical exercise during adjuvant chemotherapy | [Record](https://search.pedro.org.au/search-results/record-detail/51440) | [Full text](https://link.springer.com/article/10.1007/s10198-017-0936-0) |
| 42 | Four-year effects of exercise on fatigue and physical activity in patients with cancer | [Record](https://search.pedro.org.au/search-results/record-detail/53822) | [Full text](https://bmcmedicine.biomedcentral.com/articles/10.1186/s12916-018-1075-x) |
| 43 | Effect of low-intensity physical activity and moderate- to high-intensity physical exercise during adjuvant chemotherapy on physical fitness, fatigue, and chemotherapy completion rates: results of the PACES randomized clinical trial | [Record](https://search.pedro.org.au/search-results/record-detail/43011) | [Full text](https://ascopubs.org/doi/10.1200/JCO.2014.59.1081) |
| 44 | Impact of a home-based walking intervention on outcomes of sleep quality, emotional distress, and fatigue in patients undergoing treatment for solid tumors [with consumer summary] | [Record](https://search.pedro.org.au/search-results/record-detail/35914) | [Full text](https://academic.oup.com/oncolo/article/18/4/476/6398746?login=false) |
| 45 | Feasibility and acceptability of home-based strength training in endometrial cancer survivors | [Record](https://search.pedro.org.au/search-results/record-detail/72137) | [Full text](https://link.springer.com/article/10.1007/s11764-021-00990-3) |
| 46 | Fitbit use and activity levels from intervention to 2 years after: secondary analysis of a randomized controlled trial | [Record](https://search.pedro.org.au/search-results/record-detail/71367) | [Full text](https://mhealth.jmir.org/2022/6/e37086) |
| 47 | Aerobic exercise during chemotherapy infusion for cancer treatment: a novel randomised crossover safety and feasibility trial | [Record](https://search.pedro.org.au/search-results/record-detail/57692) | [Full text](https://link.springer.com/article/10.1007/s00520-019-04871-5) |
| 48 | Nordic pole walking for individuals with cancer: a feasibility randomized controlled trial assessing physical function and health-related quality of life | [Record](https://search.pedro.org.au/search-results/record-detail/60690) | [Abstract](https://journals.lww.com/rehabonc/abstract/2020/04000/nordic_pole_walking_for_individuals_with_cancer__a.5.aspx) |
| 49 | Effect of a technology-supported physical activity intervention on health-related quality of life, sleep, and processes of behavior change in cancer survivors: a randomized controlled trial | [Record](https://search.pedro.org.au/search-results/record-detail/62264) | [Full text](https://onlinelibrary.wiley.com/doi/10.1002/pon.5524) |
| 50 | Outcomes of a text message, Fitbit, and coaching intervention on physical activity maintenance among cancer survivors: a randomized control pilot trial [with consumer summary] | [Record](https://search.pedro.org.au/search-results/record-detail/62276) | [Full text](https://link.springer.com/article/10.1007/s11764-019-00831-4) |
| 51 | Effect of a mixed-exercise program on physical capacity and sedentary behavior in older adults during cancer treatments | [Record](https://search.pedro.org.au/search-results/record-detail/59998) | [Full text](https://link.springer.com/article/10.1007/s40520-018-1097-4) |
| 52 | Effects of exercise and cardiorespiratory fitness on estrogen metabolism in postmenopausal women | [Record](https://search.pedro.org.au/search-results/record-detail/59108) | [Full text](https://aacrjournals.org/cebp/article/27/12/1480/71447/Effects-of-Exercise-and-Cardiorespiratory-Fitness) |
| 53 | The Yale fitness intervention trial in female cancer survivors: cardiovascular and physiological outcomes | [Record](https://search.pedro.org.au/search-results/record-detail/51044) | [Full text](https://www.heartandlung.org/article/S0147-9563(17)30076-6/fulltext) |
| 54 | Social cognitive theory mediators of physical activity in a lifestyle program for cancer survivors and carers: findings from the ENRICH randomized controlled trial | [Record](https://search.pedro.org.au/search-results/record-detail/45788) | [Full text](https://ijbnpa.biomedcentral.com/articles/10.1186/s12966-016-0372-z) |
| 55 | A self-regulation-based intervention to increase physical activity in cancer patients | [Record](https://search.pedro.org.au/search-results/record-detail/46945) | [Full text](https://www.tandfonline.com/doi/full/10.1080/13548506.2015.1081255) |
| 56 | Mediators of physical exercise for improvement in cancer survivors' quality of life | [Record](https://search.pedro.org.au/search-results/record-detail/39114) | [Full text](https://onlinelibrary.wiley.com/doi/10.1002/pon.3428) |
| 57 | The role of physical activity and physical fitness in postcancer fatigue: a randomized controlled trial | [Record](https://search.pedro.org.au/search-results/record-detail/36567) | [Full text](https://link.springer.com/article/10.1007/s00520-013-1784-9) |
| 58 | Efficacy of multimodal exercise-based rehabilitation on physical activity, cardiorespiratory fitness, and patient-reported outcomes in cancer survivors: a randomized, controlled trial | [Record](https://search.pedro.org.au/search-results/record-detail/36880) | [Full text](https://www.annalsofoncology.org/article/S0923-7534(19)36931-5/fulltext) |
| 59 | Reach out to enhance wellness home-based diet-exercise intervention promotes reproducible and sustainable long-term improvements in health behaviors, body weight, and physical functioning in older, overweight/obese cancer survivors | [Record](https://search.pedro.org.au/search-results/record-detail/32862) | [Full text](https://ascopubs.org/doi/10.1200/JCO.2011.40.0895) |
| 60 | Effects of remotely-delivered physical activity education on exercise beliefs and intentions of active and nonactive cancer survivors and their partners | [Record](https://search.pedro.org.au/search-results/record-detail/72557) | [Full text](https://academic.oup.com/tbm/article-abstract/12/5/663/6582709?redirectedFrom=fulltext&login=false) |
| 61 | A randomized, feasibility trial of an exercise and nutrition-based rehabilitation programme (ENeRgy) in people with cancer | [Record](https://search.pedro.org.au/search-results/record-detail/67500) | [Full text](https://onlinelibrary.wiley.com/doi/10.1002/jcsm.12806) |
| 62 | Cancer survivors exercise at higher intensity in outdoor settings: the GECCOS trial | [Record](https://search.pedro.org.au/search-results/record-detail/67591) | [Full text](https://onlinelibrary.wiley.com/doi/10.1002/pbc.28850) |
| 63 | Effect of self-regulatory behaviour change techniques and predictors of physical activity maintenance in cancer survivors: a 12-month follow-up of the Phys-Can RCT | [Record](https://search.pedro.org.au/search-results/record-detail/68183) | [Full text](https://bmccancer.biomedcentral.com/articles/10.1186/s12885-021-08996-x) |
| 64 | Two-year follow-up of the OptiTrain randomised controlled exercise trial | [Record](https://search.pedro.org.au/search-results/record-detail/56564) | [Full text](https://link.springer.com/article/10.1007/s10549-019-05204-0) |
| 65 | Effect of a 12-week online walking intervention on health and quality of life in cancer survivors: a quasi-randomized controlled trial | [Record](https://search.pedro.org.au/search-results/record-detail/54869) | [Full text](https://www.mdpi.com/1660-4601/15/10/2081) |
| 66 | Recruitment, adherence, and retention of endometrial cancer survivors in a behavioural lifestyle programme: the Diet and Exercise in Uterine Cancer Survivors (DEUS) parallel randomised pilot trial [with consumer summary] | [Record](https://search.pedro.org.au/search-results/record-detail/51364) | [Full text](https://bmjopen.bmj.com/content/7/10/e018015) |
| 67 | Impact of a nutrition and physical activity intervention (ENRICH: Exercise and Nutrition Routine Improving Cancer Health) on health behaviors of cancer survivors and carers: a pragmatic randomized controlled trial | [Record](https://search.pedro.org.au/search-results/record-detail/44395) | [Full text](https://bmccancer.biomedcentral.com/articles/10.1186/s12885-015-1775-y) |
| 68 | Impact of a telephone-based physical activity intervention upon exercise behaviors and fitness in cancer survivors enrolled in a cooperative group setting | [Record](https://search.pedro.org.au/search-results/record-detail/31387) | [Full text](https://link.springer.com/article/10.1007/s10549-011-1882-7) |
| 69 | Feasibility of implementing physical activity behavior change counseling in an existing cancer-exercise program | [Record](https://search.pedro.org.au/search-results/record-detail/68572) | [Full text](https://www.mdpi.com/1660-4601/18/23/12705) |
| 70 | Telehealth applied to physical activity during cancer treatment: a feasibility, acceptability, and randomized pilot study | [Record](https://search.pedro.org.au/search-results/record-detail/54605) | [Full text](https://link.springer.com/article/10.1007/s00520-018-4191-4) |
| 71 | Maintenance of lifestyle changes at 12-month follow-up in a nutrition and physical activity trial for cancer survivors | [Record](https://search.pedro.org.au/search-results/record-detail/51383) | [Full text](https://www.ingentaconnect.com/content/png/ajhb/2017/00000041/00000006/art00012;jsessionid=2gh8l7pg3jvug.x-ic-live-01) |
| 72 | Results of DUET: a web-based weight loss randomized controlled feasibility trial among cancer survivors and their chosen partners | [Record](https://search.pedro.org.au/search-results/record-detail/73835) | [Full text](https://www.mdpi.com/2072-6694/15/5/1577) |
| 73 | Optimal timing of a physical exercise intervention to improve cardiorespiratory fitness: during or after chemotherapy | [Record](https://search.pedro.org.au/search-results/record-detail/72807) | [Full text](https://www.sciencedirect.com/science/article/pii/S2666087322003866?via%3Dihub) |
